# Electronic device use and beverage related sugar and caffeine intake in US adolescents

**DOI:** 10.1371/journal.pone.0223912

**Published:** 2019-10-22

**Authors:** Kelly M. Bradbury, Ofir Turel, Katherine M. Morrison

**Affiliations:** 1 Department of Pediatrics, Centre for Metabolism, Obesity and Diabetes Research, McMaster University, Hamilton, Ontario, Canada; 2 Department of Information Systems and Decision Sciences, Mihaylo College of Business and Economics, California State University—Fullerton, Fullerton, California, United States of America; University of Michigan, UNITED STATES

## Abstract

**Background:**

Despite recent declines in consumption of sugary beverages, energy drinks (ED) and sodas continue to contribute a substantial amount of sugar and caffeine to the diet of youth. Consumption of these beverages has been linked with electronic device use, however in-depth associations between sugar and caffeine intake from energy drinks and sodas with various electronic devices are not clear.

**Objective:**

Describe the relationship of soda and energy drink consumption and associated added sugar and caffeine intake with electronic device use among adolescents.

**Methods:**

Secondary data from the 2013–2016 cycles of Monitoring the Future Survey, a national, repeated, cross-sectional study, were analyzed. Information on energy drink and soda consumption by students in grades 8 and 10 (n = 32,418) from 252–263 schools randomly sampled from all US states was used.

**Results:**

Soda and energy drink consumption decreased each year from 2013–2016 while daily use of electronic devices remained stable. An additional hour/day of TV was linked to a 6.92g (6.31,7.48; p<0.001) increase in sugar intake and a 32% (OR = 1.32; 1.29,1.35; p < .001) higher risk of exceeding World Health Organization (WHO) recommended sugar intakes. Further, each hour/day of TV was linked to a 28% increased risk of exceeding caffeine recommendations (OR = 1.25–1.31; p<0.001). Each hour per day talking on a cellphone was associated with an increased risk of exceeding WHO sugar and caffeine intakes by 14% (OR = 1.11–1.16; p<0.001) and 18% (OR = 1.15–1.21; p<0.001) respectively. Video game use was only weakly linked to caffeine intake. Computer use for school was associated with lower likelihood of exceeding sugar intake cut-offs.

**Conclusion:**

While a trend towards reduced energy drink and soda intake from 2013–2016 was evident, greater electronic device use, especially TV time, was linked to higher intake of beverage-derived added sugar and caffeine amongst adolescents. Addressing these behaviours through counselling or health promotion could potentially help to reduce excess sugar and caffeine intake from sodas and energy drinks among this population.

## Introduction

Consumption of sugar-sweetened beverages (SSB) such as sodas is linked to adverse health consequences including obesity [1–3] and various metabolic health impacts [4, 5] including dyslipidemia [6], diabetes [7], dental caries [8] and poor sleep hygiene [9, 10] . Artificially-sweetened beverages are also associated with higher energy intake despite their calorie-free properties [11], possibly due to their heightened sweetness which may impact one’s perception of energy intake [12]. It is recommended that SSB and artificially-sweetened beverage intake be limited among youth [11, 13, 14] given these associated health impacts.

Among SSB, energy drinks contain high amounts of added sugar or artificial sweeteners and typically more caffeine than sodas [15]. Consumption of excess caffeine is associated with adverse health impacts including difficulties with sleep, headaches, elevated blood pressure, nausea, vomiting, diarrhea and chest pain [15, 16]. Consequently, both the American Academy of Pediatrics [17] and the Canadian Pediatric Society [15] have released position statements which urge pediatricians to educate youth and families on the potential risks of these beverages and recommend against consumption of these drinks.

SSB [18, 19] and artificially-sweetened beverage intakes have declined since the early 2000’s [19] and energy drink consumption has remained relatively stable among children and adolescents [20]. Despite these declines, a recent review of large national cohort studies found that SSB, including beverages such as energy drinks and sodas, remain a top contributor to daily energy intake among children and adolescents aged 2–19 years, with an estimated 130 calories consumed per day from these beverages [19]. Given that both the World Health Organization (WHO) [21] and the Dietary Guidelines for Americans 2015–2020 [14] recommend that no more than 10% of energy intake should come from added sugars in food or beverages, sodas and energy drinks are important sources of added sugar to consider. Furthermore, investigations on caffeine intake from beverage sources among youth demonstrate that energy drinks generally contribute approximately 5–7% of the total daily caffeine intake of adolescents and young adults, while carbonated drinks remain a top contributor to total daily caffeine intake for children and adolescents at 17% [20]. Thus, consuming these beverages may increase the likelihood of failing WHO recommendations which can lead to adverse health outcomes including dental caries and obesity [21, 22].

Youth generally report consuming energy drinks for their taste and their stimulatory effect [16], and some have hypothesized that energy drink consumption may explain the association of video game usage and poorer sleep hygiene [10, 16, 23]. Soda and energy drink consumption may also be influenced by advertisements online [24, 25] and distracted eating; something that can occur during electronic device usage. These result in adverse impacts on overall diet quality and energy intake in children and adults [26, 27]. Adults consume a greater caloric intake from snacking while watching TV alone compared to when they are socializing. Further, a randomized crossover trial in adolescent boys demonstrated that those who played video games for one hour had a significantly higher energy intake from ad libitum lunches provided afterwards compared to those who spent time resting for one hour [26, 28]. While the latter study measured energy intake after video game use, multiple observational studies have also associated energy drink and SSB intake with increased time spent on electronics [29, 30], including video games [31–33] and television watching [23, 31, 32, 34–36]. Given that SSB, including sodas and energy drinks continue to contribute a substantial amount of energy and caffeine to the youth diet, investigating the link between their consumption with multiple forms of electronic device usage in a large national representative population may help establish a deeper understanding of this relationship.

The objective of this study was to examine potential links between energy drink and soda consumption with use of electronic devices amongst adolescents. Due in part to the distracting properties of electronic device use, we hypothesized that caffeine and added sugar intakes from beverage sources would be directly associated with electronic device use time. Additionally, given the needed stimulation and concentration for video gaming, and marketing campaigns that target video gamers, we expected a particularly strong association between energy drink and caffeine intake with video game usage.

## Methods

### Study population

Participants were students in grades 8 and 10 who completed the 2013–2016 cycles of the Monitoring the Future study [37, 38]. The Monitoring the Future project relies on anonymized survey data to assist policymakers in understanding youth attitudes, beliefs and behaviours including alcohol and drug use (Funded by the National Institutes of Health; more information available at: http://www.monitoringthefuture.org/purpose.html). The current analysis is a secondary analysis of fully anonymized data made available from the Monitoring the Future Study through an application process with co-author OT. The Monitoring the Future study has an application process for secondary data analysis on de-identified data that does not require additional reviews by ethics boards outside of the IRB at Michigan.

The Monitoring the Future study is carried out in schools, is voluntary and parents and students are given the opportunity to decline participation. All procedures are reviewed and approved on an annual basis by the University of Michigan’s Institutional Review Board for compliance with federal guidelines for the treatment of human subjects [38]. This study is based on a survey, is nationally representative and incorporates repeated cross-sectional studies in youth (i.e. randomly sampled schools each year). Details of the study conduct are reported elsewhere [39]. Data from 252–263 participating schools from 2013 through 2016 were used, and the annual population sizes varied from 15,015–17,643 students in 8^th^ grade (13–14 years old) and 13,262–16,147 students in 10^th^ grade (15–16 years old). Depending on the cycle year, response rates varied between 87% and 90% [38].

The full sample included 152,172 responses but different sections of the survey were completed by each school. Consequently, there were 33,261 responses from schools that completed all components of the survey relevant to the current analysis. From these respondents, we removed all records with missing values (843; 2.53%). The retained analytical sample included 32,418 records. Given the small percentage of missing values, it is unreasonable that omitting these records have skewed the results.

### Study measures

The primary outcomes were daily servings of energy drinks and shots (referred to as energy drinks herein) and sodas (diet, regular); and estimated daily sugar (g/day) and caffeine (mg/day) consumption based on self-reported intake of 12-ounce units (1 serving) of energy drinks, energy shots, sugar-sweetened sodas and diet sodas. Daily beverage intake was collected as part of the Monitoring the Future study and descriptions of each type of drink with examples were provided (S1 Table). Responses ranged from daily intake of "None", "Less than one", "One", "Two", "Three", "Four", "Five or six" or "Seven or more".

Estimated sugar (g/day) and caffeine (mg/day) intakes were calculated based on the reported number of servings and estimated average amount of either sugar or caffeine contained in each beverage type. These estimates were based on national consumer studies information [40, 41] which included data for 354 energy drinks, 87 energy shots, 83 soda drinks and 38 diet soda drinks. Using this calculation, the estimated average sugar (g) per unit was 24.91g/unit of energy drink, 1.83g/unit energy shot, 34.97g/unit of sugar-sweetened sodas and 0g/unit of diet soda. The average caffeine content per drink was estimated to be 110.05 mg/unit of energy drinks and 150.29 mg/unit of energy shots according to reported caffeine content for 20 popular energy drinks and 7 energy shots [40]. The average caffeine intake for both regular sodas and diet sodas was estimated to be 42.97 mg/unit based on reported caffeine content of 72 common soda drinks [40].

In addition to daily intake as a continuous variable, we evaluated if recommended sugar and/or caffeine intake were likely to be exceeded as a result of reported beverage intake alone. Assessing the likelihood of failing recommendations was of interest to further understand the impact of beverage consumption on sugar and caffeine intakes among adolescents. The WHO strongly recommends that energy intake from sugar should not exceed 10% of total energy intake and also suggests a conditional limit of 5% daily energy intake from added sugars [21]. We estimated the individual energy requirement for each participant using US Department of Agriculture (USDA) daily calorie needs for moderately active youth, stratified by age and sex [14], to determine if added sugar from the beverages studied exceeded 5% and 10% of estimated daily energy intake (S2 Table). As the recommended caffeine intake for adolescents is based on body weight and we did not have exact weights of participants, we extracted the weight at the 50^th^ percentile according to each sex and age group (13–14 years in 8th grade; 15–16 years in 10th grade) from the Center for Disease Control (CDC) growth charts [42]. This weight was multiplied by the recommended daily caffeine intake for adolescents (≤2.5 mg/kg) [43] to generate the caffeine threshold for each age and sex.

Electronic device use was based on self-report responses of the number of hours per week of 1) computer use for school, 2) videogames use, 3) social media use, 4) watching TV (weighted average of hours per weekday and weekend day), and 5) talking on cellphone (no texting, social media use, etc.). The questionnaires included a range of responses (“None," “less than 1 hour,” “1–2 hours,” “3–5 hours,” “6–9 hours,” “10–19 hours,” 20–29 hours,” “30–39 hours,” or "40 or more" per week), which were transformed by using the mid-point for closed ranges, or the lowest value for open ranges. Weekly values were converted to daily values through division by seven.

Covariates included in the analysis of the relationship between sugar or caffeine consumption and electronic device use were: year of survey administration, grade (8^th^ grade = 0), sex (female = 0), self-reported parental education as an indicator of socioeconomic status and hours spent unattended at home, a reported risk factor for unhealthy and risky behaviours [44]. Parental education level responses were “Completed grade school or less”, “Some high school”, “Completed high school”, “Some college”, “Completed college”, “Graduate or professional school after college”. Responses were transformed into a scale from 1–6 respectively. Survey questions with variable names, respective anchors and methods of transformation are included in S1 Table.

### Statistical analysis

All analyses were performed in SPSS 25. Differences in the proportions of youth exceeding recommendations between 2013 and 2016 were analyzed using chi-square testing. All models were estimated with bootstrapping with 2,000 re-samples for generating bias-corrected 95% confidence intervals for estimates to avoid distributional assumptions, improve robustness [45] and to allow the statistical comparison of regression coefficients [46]. Regression analyses were used to predict daily caffeine and sugar intakes from the examined drinks, with daily hours of electronic device use and the aforementioned covariates as predictors. Logistic regression models were used to determine the relationship of hours of daily electronic device use with whether or not the child exceeded recommended sugar and caffeine intake from soda and ED, after controlling for covariates. For the odds ratios (OR), non-overlapping confidence intervals or distances from upper/lower bound to the estimate point that had <50% overlap was deemed indicative of significant (p<0.05) differences between the coefficients [46].

## Results

### Sample characteristics and beverage intake trends

The analytical sample (n = 32,418) included 15,834 students in grade 8 (48.8%) and 16,584 in grade 10 (51.2%) who responded to all questions regarding daily electronic device use and soda and energy drink consumption. Participants were nearly equal male (48.1%; n = 15,593) and female (**Table 1)** and 13.9% were Black; 64.8% White and 21.3% Hispanic.

**Table 1:** Sample population from each survey year (total n = 32,418), and home characteristics.

	Male (n = 15,593)		Female (n = 16,825)		All (n = 32,418)
	8^th^ Grade (n = 7534)	10^th^ Grade (n = 8059)	8^th^ Grade (n = 8300)	10^th^ Grade (n = 8525)	
**Year (n (valid %))**					
2013	1,838 (24.4%)	1,885 (23.4%)	1,974 (23.8%)	2,019 (23.7%)	7,716 (23.8%)
2014	1,746 (23.2%)	1,891 (23.5%)	2,034 (24.5%)	1,960 (23.0%)	7,631 (23.5%)
2015	1,802 (23.9%)	2,263 (28.1%)	2,008 (24.2%)	2,330 (27.3%)	8,403 (25.9%)
2016	2,148 (28.5%)	2,020 (25.1%)	2,284 (27.5%)	2,216 (26.0%)	8,668 (26.7%)
**Home Environment (mean, (SD))**					
Mother Education Scale	4.29 (1.36)	4.33 (1.32)	4.16 (1.41)	4.23 (1.38)	4.25 (1.37)
Father Education Scale	4.06 (1.42)	4.13 (1.40)	3.93 (1.44)	4.00 (1.44)	4.03 (1.43)
Hours alone after school (h/day)	1.41 (1.49)	1.50 (1.51)	1.38 (1.51)	1.52 (1.55)	1.45 (1.52)

* Parental Education Scale: 1 = Completed grade school or less, 2 = Some high school, 3 = Completed high school, 4 = Some college, 5 = Completed college, 6 = Graduate or professional school after college.

**Fig 1:**
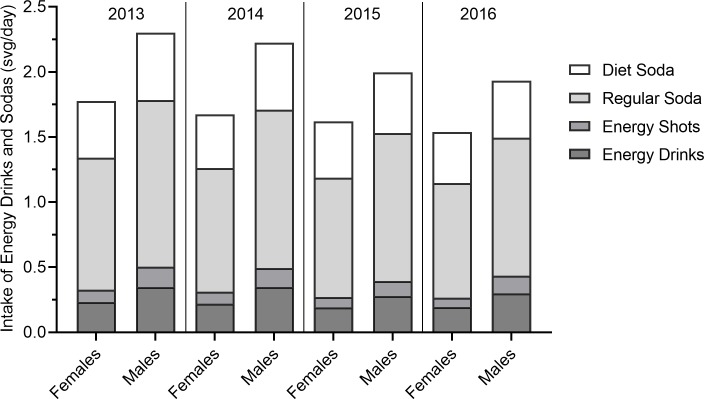
Average daily intake of energy drinks and soda (servings per day) by year and sex.

**Fig 2:**
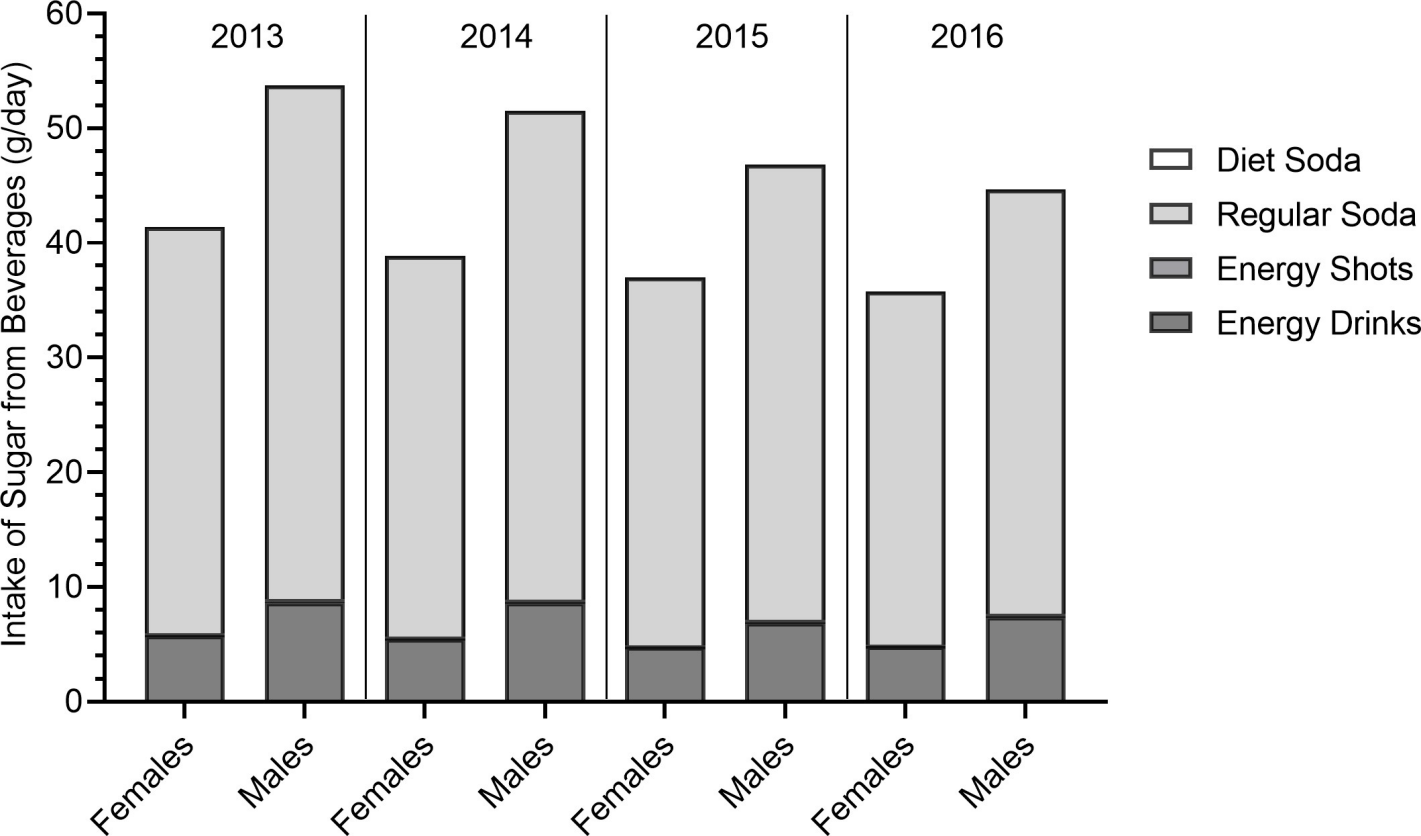
Daily sugar intake attributable to consumption of energy drinks and sodas among males and females from 2013–2016.

**Fig 3:**
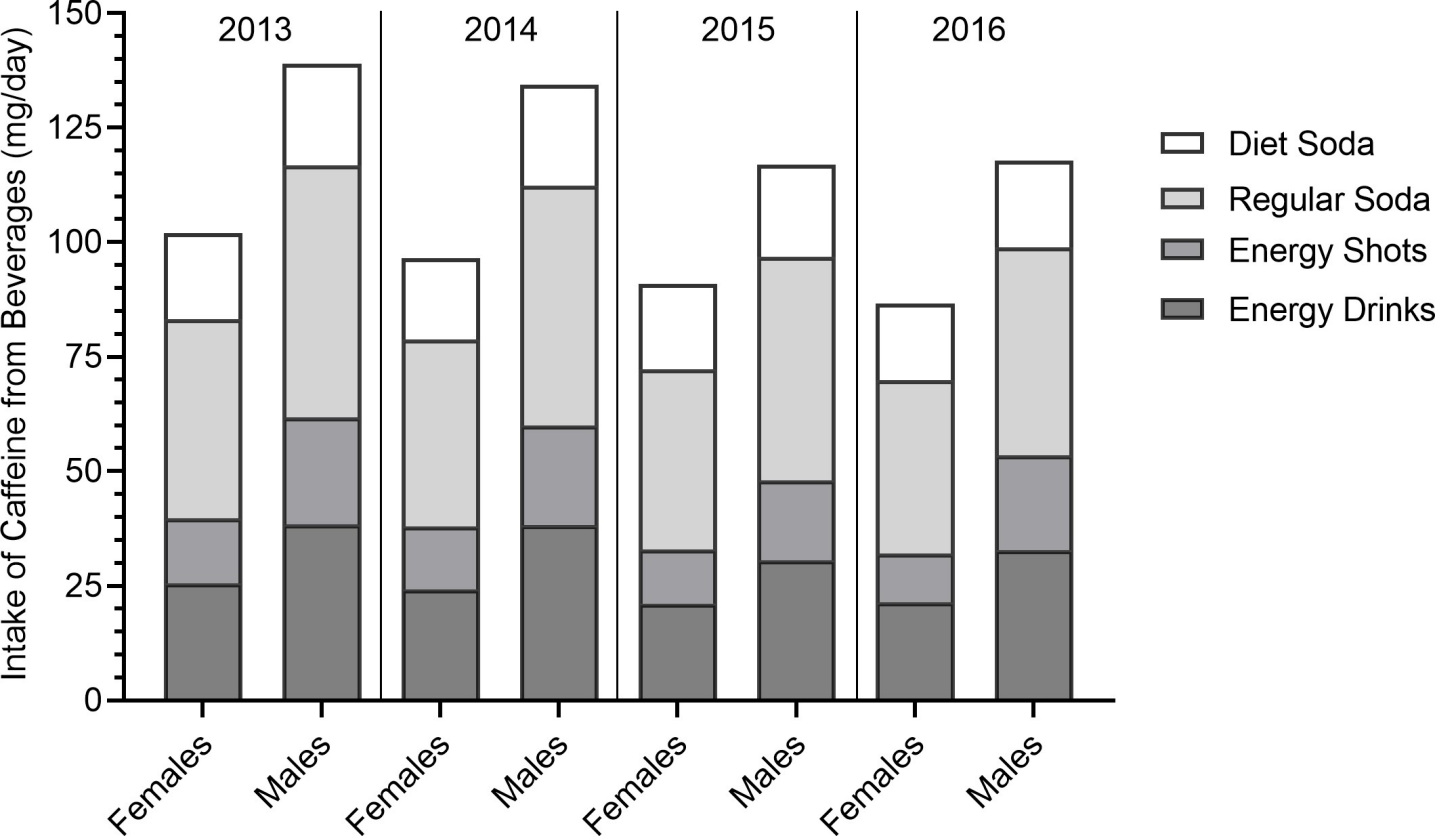
Daily caffeine intake attributable to consumption of energy drinks and sodas among males and females from 2013–2016.

Reported consumption of energy drinks, sodas and beverage-related sugar and caffeine intake declined each year from 2013 to 2016 (**Figs 1–3; Table 2).** Accordingly, the number of youth who exceeded conditional (5% of energy intake) and strong (10% of energy intake) recommendations for sugar intake and recommendations for caffeine intake (<2.5mg/kg) as a result of energy drink and soda consumption also declined. In 2013, 53.8% of males and 45.5% of females exceeded the conditional sugar intake recommendations and 32.2% of males and 25.7% of females exceeded the strong sugar intake recommendations. In 2016, the frequency of exceeding conditional recommendations decreased to 47.0% among males and 41.6% among females while 26.0% of males and 21.6% of females exceeded the strong recommendations. Declines were particularly notable for males in Grade 8. Girls in Grade 10 reported declines in the more stringent limit of added glucose intake only. In 2013, 26.0% of youth exceeded recommended caffeine intake from soda energy drink consumption compared to 21.2% in 2016. This decline was notable across sex and grade level, but was not significant in girls in Grade 10. Thus, the largest decline in exceeding recommended sugar and caffeine intake thresholds was seen in males in Grade 8 and these declines over time were less evident in the older girls.

**Table 2:** Frequency of youth who exceed recommended sugar and caffeine intakes from energy drinks and sodas during 2013–2016 (n = 32,418) by sex, grade and year.

	Male (n = 15,593)		Female (n = 16,825)	
	8^th^ Grade (n = 7534)	10^th^ Grade (n = 8059)	8^th^ Grade (n = 8300)	10^th^ Grade (n = 8525)
**Percentage of youth who exceed recommended 5% energy intake from added sugars in reported beverages (n, (valid %))**				
**2013**	1113 (60.55%)	891 (47.27%)	1012 (51.27%)	803 (39.77%)
**2014**	1012 (57.96%)	895 (47.33%)	1010 (49.66%)	739 (37.70%)
**2015**	1024 (56.83%)	928 (41.01%)	949 (47.26%)	824 (35.36%)
**2016**	1100 (51.21%)	858 (42.48%)	1029 (45.05%)	844 (38.09%)
**Total**	4249 (56.40%)	3572 (44.32%)	4000 (48.19%)	3210 (37.65%)
Change from 2013 to 2016*	-9.34% (p<0.0001)	-4.79% (p = 0.0026)	-6.22% (p = 0.0001)	-1.68% (p = 0.2627)
**Percentage of youth who exceed recommended 10% energy intake from added sugars in reported beverages (n, (valid %))**				
**2013**	674 (36.67%)	524 (27.80%)	592 (29.99%)	436 (21.59%)
**2014**	617 (35.34%)	497 (26.28%)	570 (28.02%)	381 (19.44%)
**2015**	632 (35.07%)	520 (22.98%)	529 (26.34%)	422 (18.11%)
**2016**	612 (28.49%)	470 (23.27%)	565 (24.74%)	407 (18.37%)
**Total**	2535 (33.65%)	2011 (24.50%)	2256 (27.18%)	1646 (19.31%)
Change from 2013 to 2016*	-8.18% (p<0.0001)	-4.53% (p = 0.0012)	-5.25% (p = 0.0001)	-3.22% (p = 0.0088)
**Percentage of youth who exceed recommended caffeine intake from reported beverages** **(n, (valid %))**				
**2013**	685 (37.27%)	433 (22.97%)	582 (29.48%)	350 (18.57%)
**2014**	624 (35.74%)	415 (21.95%)	573 (28.17%)	299 (15.81%)
**2015**	602 (33.41%)	407 (17.98%)	527 (26.25%)	340 (15.02%)
**2016**	628 (29.24%)	385 (19.06%)	554 (24.26%)	332 (16.49%)
**Total**	2539 (33.70%)	1640 (20.35%)	2236 (26.94%)	1322 (15.51%)
Change from 2013 to 2016*	-8.03% (p<0.0001)	-3.91% (p = 0.0027)	-5.22% (p = 0.0001)	-2.08% (p = 0.0751)

* Cells report the chi-square test results for difference in proportions

### Electronic device use and estimated daily sugar and caffeine intake from sodas and ED

**Fig 4:**
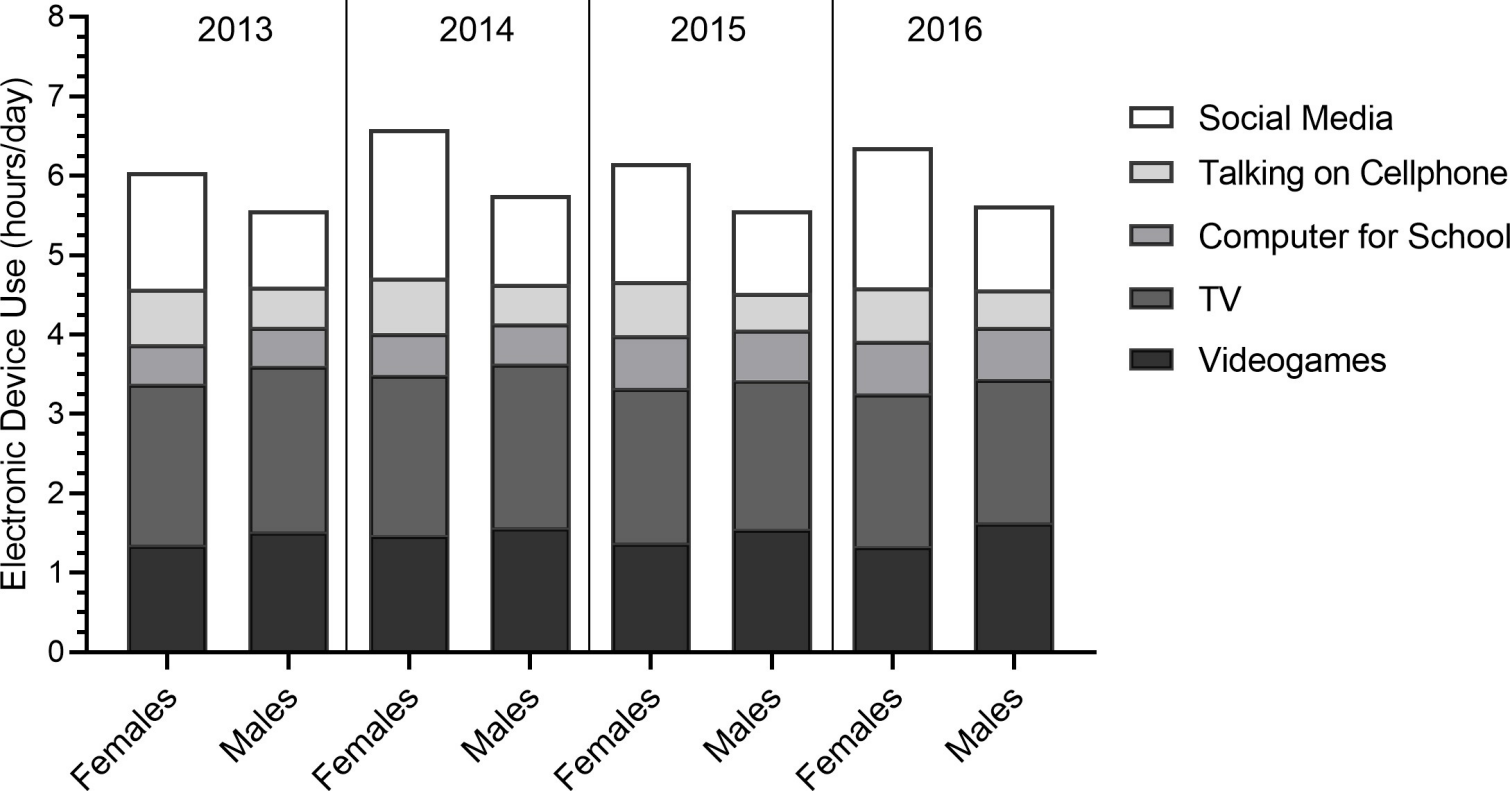
Reported electronic device use (hours per day) among males and females from 2013–2016*. * Note that multi-tasking is possible (i.e. use more than one device at once) and therefore cumulative hours of electronic device use should be interpreted with caution.

On average, these youth used their devices as follows; 1.97 h/day (SD = 1.36) on TV, 1.48 h/day (SD = 1.82) on video games, 1.36 h/day (SD = 1.74) on social media, 0.59 h/day (SD = 1.26) talking on the cellphone and 0.58 h/day (SD = 1.01) on computer use for school (Fig 4). Reported use of electronic devices was higher among females compared to males throughout the study duration.

Reported use of all electronic devices except ‘computer use for school’ was related to higher estimated daily sugar and caffeine intake, independent of grade and sex of the participant, parental education and hours spent unattended at home. Daily sugar intake was 6.92g (6.31, 7.48; p< 0.001) higher for each hour spent watching TV, 5.56g (4.68, 6.50; p< 0.001) higher per hour talking on the phone, 1.99g (1.54, 2.46; p< 0.001) higher per hour spent playing video-games and 1.65g (1.13, 2.14; p< 0.001) higher for each additional hour of social-media use (**Table 3**). When considering the daily use of each electronic device and the associated increase in sugar consumption per hour of device use, TV use was associated with an additional 14g of sugar per day, while videogame use, talking on a cellphone and social media use were, together, associated with an additional 8.2g of added sugar intake each day.

**Table 3:** Regression models of caffeine and sugar intakes and associations with electronic device use.

	Estimated Daily Sugar Intake (g) from Sodas and Energy Drinks		Estimated Daily Caffeine Intake (mg) Sodas and Energy Drinks	
Controls	β (P)	95% CI	β (P)	95% CI
Year of Administration	-1.98 (p< 0.001)	[-2.55; -1.42]	-5.54 (p< 0.001)	[-7.56; -3.51]
Grade (0 = 8th, 1 = 10th)	-8.88 (p< 0.001)	[-10.21; -7.59]	-31.09 (p<0.001)	[-35.83; -26.44]
Sex (0 = female, 1 = male)	13.80 (p<0.001)	[12.49; 15.13]	41.23 (p< 0.001)	[36.70; 45.74]
Mother Education	-2.27 (p< 0.001)	[-2.87; -1.64]	-6.62 (p< 0.001)	[-8.94; -4.48]
Father Education	-3.41 (p< 0.001)	[-3.95; -2.85]	-9.27 (p< 0.001)	[-11.11; -7.31]
Hours Alone After School	2.67 (p< 0.001)	[2.16; 3.19]	10.80 (p< 0.001)	[8.99; 12.70]
**Electronic Device Use**				
Computer Use for School (h/Day)	0.36 (p< 0.261)	[-0.42; 1.22]	7.14 (p< 0.001)	[3.65; 11.21]
Videogame Use (h/Day)	1.99 (p< 0.001)	[1.54; 2.46]	2.78 (p< 0.001)	[1.09; 4.40]
Social Media Use (h/Day)	1.65 (p< 0.001)	[.13; 2.14]	5.21 (p< 0.001)	[3.51; 6.99]
TV Watching (h/Day)	6.92 (p< 0.001)	[6.31; 7.48]	16.92 (p< 0.001)	[14.65; 19.03]
Talking on Cellphone (h/Day)	5.56 (p< 0.001)	[4.68; 6.50]	21.86 (p< 0.001)	[18.25;25.81]
**R**^**2**^	13.1%		10.0%	

Caffeine intake from beverages was also higher with increasing electronic device use. Caffeine intake was 7.14mg (3.65, 11.21 p< 0.001) higher/ hour of computer use for school, 2.78mg (1.09, 4.40; p< 0.001) higher/hour of video games, 5.21mg (3.51, 6.99; p< 0.001) higher/hour of social media use, 16.92mg (14.65, 19.03; p< 0.001) higher/hour watching television, and 21.86mg (18.25, 25.81; p< 0.001) higher/hour talking on the phone. Considering device use and additional intake per hour of use, TV watching was associated with intake of 32 mg/day caffeine while video gaming was associated with estimated intake of only 4.4 additional mg caffeine/day.

### Use of electronic devices and the likelihood of exceeding recommended added sugar and caffeine intakes

#### Sugar intake

Grade level, parental education and sex were independently related to the risk of exceeding added sugar intake recommendations. Similar associations were found for both the strong (10%) sugar intake recommendation (**Table 4**) and the conditional (5%) sugar recommendation (**Table 5**), however the strong recommendation demonstrated more significant associations with screen use. The likelihood of exceeding the strong (10%) sugar intake recommendations (**Table 4**) was seven percent higher with each hour of social media and video game use (1.05–1.09; p<0.001), 32 percent higher per hour of TV watching (1.29–1.35; p<0.001) and 15% higher for each hour spent talking on the cellphone (1.14–1.20; p<0.001). Considering that students spent most of their electronic device use time watching TV and that each hour of TV was associated with the highest likelihood of exceeding sugar recommendations, it is evident that time spent watching TV had the greatest influence on the likelihood of exceeding the recommendations for added sugar intake from the beverages studied.

**Table 4:** Logistic regression models of the relationship of electronic device use to the likelihood of exceeding strong (10%) sugar intake recommendations through beverage consumption.

	Exceeds Recommended Sugar Intake (10%)		
Controls	β (P)	OR	95% CI
Year of Administration	-0.08 (p<0.001)	0.92	[0.90;0.95]
Grade (0 = 8th, 1 = 10th)	-0.4 (p<0.001)	0.67	[0.63;0.71]
Sex (0 = female, 1 = male)	0.48 (p<0.001)	1.62	[1.53;1.72]
Mother education	-0.11 (p<0.001)	0.90	[0.88;0.92]
Father Education	-0.16 (p<0.001)	0.85	[0.83;0.87]
Hours alone after school	0.09 (p<0.001)	1.10	[1.08;1.12]
**Electronic Device Use**			
Computer Use for School (h/day)	-0.05 (p<0.005)	0.95	[0.92;0.98]
Videogames Use (h/day)	0.07 (p<0.001)	1.07	[1.05;1.09]
Social Media Use (h/day)	0.07 (p<0.001)	1.07	[1.05;1.09]
TV Watching (h/day)	0.28 (p<0.001)	1.32	[1.29;1.35]
Talking on Cellphone (h/day)	0.15 (p<0.001)	1.17	[1.14;1.20]
Cox & Snell R^2^	10.9%		
Nagelkerke R^2^	14.6%		

**Table 5:** Logistic regression models of the relationship of electronic device use and the likelihood of exceeding conditional (5%) sugar intake recommendations through beverage consumption.

	Exceeds Recommended Sugar Intake (5%)		
Controls	β (P)	OR	95% CI
Year of Administration	-0.06 (p<0.001)	0.94	[0.92;0.96]
Grade (0 = 8th, 1 = 10th)	-0.42 (p<0.001)	0.66	[0.63;0.69]
Sex (0 = female, 1 = male)	0.46 (p<0.001)	1.59	[1.51;1.68]
Mother education	-0.09 (p<0.001)	0.91	[0.90;0.93]
Father Education	-0.17 (p<0.001)	0.84	[0.83;0.86]
Hours alone after school	0.07 (p<0.001)	1.07	[1.05;1.09]
**Electronic Device Use**			
Computer Use for School (h/day)	-0.07 (p < .001)	0.93	[0.91;0.96]
Videogames Use (h/day)	0.06 (p < .001)	1.06	[1.05;1.08]
Social Media Use (h/day)	0.07 (p < .001)	1.07	[1.05;1.09]
TV Watching (h/day)	0.27 (p < .001)	1.31	[1.28;1.34]
Talking on Cellphone (h/day)	0.13 (p < .001)	1.14	[1.11;1.16]
Cox & Snell R^2^	10.2%		
Nagelkerke R^2^	15.6%		

### Caffeine intake

Daily usage times of all electronic devices except “computer use for school”, were also independently associated with increased odds for exceeding recommended daily caffeine intake from sodas and ED (Table 6). Independent of the covariates, the odds of exceeding caffeine recommendations were higher by 28% (OR = 1.25–1.31; p<0.001) per hour of television watching, 18% (OR = 1.15–1.21; p<0.001) per hour of talking on a cellphone, 9% (1.06–1.11; p<0.001) per hour of social media use, and 4% (1.02–1.06; p<0.001) per hour of video gaming.

**Table 6:** Logistic regression models assessing the OR of exceeding caffeine intake recommendations (>2.5mg/kg) with covariates and reported electronic device uses.

	Exceeds Recommended Caffeine Intake (>2.5mg/kg bodyweight)		
Controls	β (P)	OR	95% CI
Year of Administration	-0.09 (p< 0.001)	0.92	[0.90;0.94]
Grade (0 = 8th, 1 = 10th)	-0.73 (p< 0.001)	0.48	[0.45;0.52]
Sex (0 = female, 1 = male)	0.53 (p< 0.001)	1.70	[1.58;1.80]
Mother education	-0.09(p< 0.001)	0.91	[0.89;0.93]
Father Education	-0.16 (p< 0.001)	0.85	[0.83;0.88]
Hours alone after school	0.13 (p< 0.001)	1.14	[1.12;1.16]
**Electronic Device Use**			
Computer Use for School (h/Day)	-0.01 (p< 0.540)	0.99	[0.95;1.02]
Videogames Use (h/Day)	0.04 (p< 0.001)	1.04	[1.02;1.06]
Social Media Use (h/Day)	0.08 (p< 0.001)	1.09	[1.06;1.11]
TV Watching (h/Day)	0.25 (p< 0.001)	1.28	[1.25;1.31]
Talking on Cellphone (h/Day)	0.17 (p<0.001)	1.18	[1.15;1.21]
Cox & Snell R^2^	10.2%		
Nagelkerke R^2^	15.6%		

## Discussion

The observed decline in soda and energy drink consumption amongst youth from 2013 to 2016 observed in this study is consistent with findings from other large cohort studies [18] [19] [20]. This may be the result of government initiatives restricting the sale of SSBs and prohibiting the sale of energy drinks in schools following a statement from the AAP in 2004 [47]. However, energy intake [19] and caffeine intake [20] from these beverage sources continues to be high, with close to 50% of youth exceeding 5% of energy intake beverage-sourced sugars and 22% to 26% of females and males exceeding 10% energy from sugars.

Our primary objective was to examine the relationship of electronic device use and consumption of sugar and caffeine from soda and ED. We identified a direct relationship between sugar and caffeine intake from beverages and electronic device use. Our findings suggest that watching TV and talking on the cellphone were associated with the greatest odds of exceeding recommended intakes of sugar and caffeine. However, in contrast to our expectation, time spent video gaming contributed relatively little to added sugar and caffeine intake.

In previous studies, time spent watching TV was independently associated with SSB consumption among adolescents [32]. Additionally, in a large cross-sectional survey of grade 9–12 students in the US conducted in 2009, watching >2 hours of TV per day significantly increased the odds of consuming ≥1 energy drink and ≥1 soda per day [35]. Thus, despite the rapidly changing use patterns of electronic devices, watching TV remains an important correlate of SSB consumption. The strength of this relationship may in part due to advertising of beverages to youth [48, 49] or because both hands are free while watching TV enabling intake of food and beverages [33, 50], which may lead to poorer diet quality [51] compared to another electronic device use such as video gaming or computer use.

Few studies have reported on the relationship between SSB or energy drinks and time spent on a cellphone. One previous study has described a positive relationship between cellphone use and energy intake. However this study did not investigate talking on the phone exclusively [52]. Given that many adolescents own cellphones, further research could be conducted to better understand this relationship between cellphone use (i.e. talking) and diet quality.

The weak relationship between video game use and both sugar and caffeine intakes in the current study was surprising and counter to our original hypothesis. An increase of one hour spent playing video games increased the odds of exceeding sugar and caffeine intakes by only approximately 7%. Previous studies have demonstrated a much stronger relationship among energy drink consumption and video-gaming. Larson et al. [53] found that, among grade 6–12 students, consuming at least one energy drink per week was associated with an additional four hours of video-gaming per week [53]. Similarly, a large survey of 14–18-year-old youth in 2008 demonstrated that males and females who reported playing video games on a weekly basis were more likely to consume between one and three energy drinks per day [31]. While consumption of SSB including energy drinks has been consistently associated with greater video game use among adolescents and children [30, 33, 35, 54, 55], previous studies often do not differentiate energy drinks from other SSB beverages such as sodas, limiting our ability to compare results. [56]

Each hour of social media use is related to the risk of exceeding both caffeine and added sugar recommendations. Social media use was higher in females than males, but overall, contributed less to electronic device use time than either video games or watching TV. Although research involving social media use and beverage intake is not well-described, a previous study by Sampasa-Kayinga et al. (2015) collected social network use from nearly 10,000 adolescent students (grades 7–12) in Canada as part of a drug use and health survey. The findings demonstrated that nearly 40% of youth spent on average of 1–2 hours of social media use per day, similar to the average 1.36 daily hours reported here. When the relationship between beverage intake and social networking site use was investigated, youth who self-reported using social networking sites for two hours per day were nearly twice as likely to consume SSB in the past week and were more than three times more likely to drink an energy drink in the past year after adjusting for similar covariates (age, sex, socioeconomic status) [31, 57]. Although the average time spent on social media was similar to the current study, the relationship between energy drink and soda consumption is difficult to compare given the different time frame referred to in this previous study. However, an increased risk of consuming caffeine and sugar from beverage sources including SSB and energy drinks is evident in both studies. Given that previous research has limited focus on the relationship of social media use and nutritional behaviours, this may also highlight an area where further research is needed.

In summary, the current results demonstrate a direct relationship between use of various electronic devices and sugar and caffeine intake from beverages. These findings are important to consider given poorer diet quality and increased energy intake has also been correlated with screen time [26, 58], thought to be due in part to distracted eating and snacking [18]. A higher energy intake contributed to by added sugars found in SSB may drive weight gain [1, 2] and associated co-morbidities [59] while reducing sugar intake may help to improve weight status [60–62], diet quality [63, 64] and metabolic health[65] [66, 67]. Additionally, high reported levels of screen time in this population is also concerning given that time spent on electronic devices is reported to promote a sedentary lifestyle [33] and lower physical activity, increased anxiety and depression [68] and may also impact development in young children [69].

Identifying which types of electronic use are most strongly correlated with sugar and caffeine intake and their appropriate use among adolescents is important to help inform public health efforts to improve lifestyles among this demographic. [56]Furthermore, previous studies have shown that dietary habits are worsened when sleep quality is affected [70] potentially due to disinhibited or distracted eating and consumption of high energy foods, which has been shown to increase the risk of obesity in adults [26, 71] and children [72]. While the current findings did not assess sleep, the effect of lack of sleep on soda and energy drink consumption and electronic device use highlights a potential opportunity for further research.—especially given that energy drinks may induce increased wakefulness [16] and sleep duration is inversely related to soda consumption[73] and screen time [10, 74, 75].

The findings from this large, nationally-representative population of youth sampled from all US states and provides an increased understanding of the contribution of energy drinks and sodas relative to current recommendations. Additionally, the multiple electronic device uses reported allow for a more nuanced understanding and comparison of the relationship of technology use with sugar and caffeine intake. Further, the inclusion of multiple years has allowed study of recent trends in these two important youth behaviour areas. However, there are limitations that should be considered. The sources of the caffeine and sugar intakes were limited to soda and energy drinks and the content per drink was estimated based on average content amongst the beverage groups; and were not based on intake of specific brands. Although older students had lower caffeine intake from soda and ED, the report that more than 20% of youth exceeded recommended caffeine intake from soda and energy drink consumption may be an underestimate of overall caffeine intake as other sources of caffeine such as coffee or tea were not included [76]. Similarly, we estimated the daily energy intake based on the 50^th^ percentile weight as reported by the CDC; we were unable to apply a specific intake for each person based on their individual body weights. Thus, we have only been able to estimate the energy intake needs, and consequently the 5% and 10% threshold for added sugar that are recommended by the WHO. Multi-tasking was not directly assessed in the survey and the cumulative hours of electronic device use should be interpreted with caution, as there is the possibility that youth utilized more than one electronic device at a time. Similarly, TV use was described as total time spent watching TV, however youth may not have included TV time on multiple different mediums (e.g. laptop and online streaming). Finally, the results are based on self-reports and may be subject to recall bias. Differences between children in Grade 8 and Grade 10 from 2013–2016 were obtained comparing cross-sectional data and do not represent a decline with increasing age of the individual. Such within-individual trends cannot be assessed with our data.

## Conclusion

To conclude, a trend of reduced reported consumption of sodas and energy drinks amongst American youth from 2013–2016 was identified while electronic device use remained relatively stable. In spite of these declines, over 27% of youth exceeded strong (<10%) sugar intake recommendations and 21% exceeded recommended caffeine intakes based on soda and energy drink consumption alone in 2016. Consumption of added sugar and caffeine and risks of exceeding respective thresholds through energy drink and soda intake were higher amongst youth with greater daily usage of TV, video games, talking on the phone and social media use. Considering these associations may assist pediatricians, public health advocates and parents to develop strategies to reduce the risk of excessive intakes of added sugar and caffeine amongst youth.

## Supporting information

S1 Table Variables, respective measures and transformations used for analysis. Further information on variables is available at: https://www.icpsr.umich.edu/icpsrweb/NAHDAP/studies/37183/variables. Note that no reliability indices are available given that the questions are single items.
(DOCX)

S2 Table Estimated weights and respective caffeine and sugar intake according to age and sex. (DOCX)
