# Supplementary material for: Electronic device use and beverage related sugar and caffeine intake in US adolescents
Source: PLoS One. 2019 Oct 22;14(10):e0223912. doi: 10.1371/journal.pone.0223912 (PMC6805001; doi:10.1371/journal.pone.0223912)
Supplement: S1 Table — Further information on variables is available at: https://www.icpsr.umich.edu/icpsrweb/NAHDAP/studies/37183/variables. Note that no reliability indices are available given that the questions are single items. (DOCX) [file pone.0223912.s001.docx]

**S1 Table.** Variables, respective measures and transformations used for analysis. Further information on variables is available at: https://www.icpsr.umich.edu/icpsrweb/NAHDAP/studies/37183/variables. Note that no reliability indices are available given that the questions are single items.

| **Variable (Variable Code, if available)** | **Question** | **Anchors** | **Transformation** |
| --- | --- | --- | --- |
| **Study Variables** | | | |
| Hours Computer for School/ Day  (V7544) | About how many hours a week do you spend using a computer to do school work? | 1="None" to 9="40 or more" | Values were re-coded (e.g., “3-5 hours" was coded as 4 hours) and divided by 7 to convert to h/day. |
| Hours Videogames/ Day  (V7553) | About how many hours a week do you spend playing electronic games on a computer, TV, phone, or other device? | 1="None" to 9="40 or more" | Values were re-coded (e.g., "40 or more" was coded as 40 h/week) and divided by 7 to convert to h/day. |
| Hours Social Media/ Day  (V7589) | About how many hours a week do you spend visiting social networking websites like Facebook? | 1="None" to 9="40 or more" | Values were re-coded (e.g., "6-9 hours" was coded as 7.5 h/week) and divided by 7 to convert to h/day. |
| Hours TV/ Day  (V7325; V7326) | 1) How much TV do you estimate you watch on an average WEEKDAY?  2) How much TV do you estimate you watch on an average WEEKEND (both Saturday and Sunday combined)? | 1) 1="None" to  7="5 hours or more"  2) 1="None" to  7="9 hours or more " | Weighted average of weekday and weekend re-coded hours/day (e.g., “3-5 hours" was coded as 4 hours). |
| Hours Talking on Cellphone/ Day  (V7563) | About how many hours a week do you spend talking on a cell phone? | 1="None" to 9="40 or more" | Values were re-coded (e.g., "10-19 hours " was coded as 14.5 h/week) and divided by 7 to convert to h/day. |
| g Sugar Intake from Unhealthy Drinks / Day | 1) About how many (if any) energy drinks do you drink PER DAY, on average?  2) How many (if any) energy  drink shots do you drink PER DAY, on average?  3) How many (if any) 12-ounce cans  or bottles (or the equivalent) of regular (non- diet) soft drinks do you drink PER DAY, on average?  4) How many (if any) 12-ounce cans or bottles (or the equivalent) of diet soft drinks do you drink PER DAY, on average? | 0="None" to 7="7 or more" | For each drink type, the average grams of sugar calculated based on national samples was multiplied by the drink units/day. The estimated g sugar/day from each drink type were added to create an estimate of daily sugar intake from all four types of unhealthy drinks. |
| mg Caffeine Intake from Unhealthy Drinks / Day | Same as above | Same as above | For each drink type, the average mg of caffeine calculated based on national samples was multiplied by the drink units/day. The estimated mg caffeine/day from each drink type were added to create an estimate of daily caffeine intake from all four types of unhealthy drinks. |
| **Controls** | | | |
| Year of Administration | NA | 2013-2016 | NA |
| Grade | NA | 8th, 10th | 8th grade= 0; 10th grade=1 |
| Sex | What is your sex? | F, M | 0=female; 1=male |
| Mother Education | What is the highest level of schooling your mother completed? | 1="Completed grade school or less", 2=” Some high school", 3="Completed high school", 4= “Some college", 5="Completed college", and 6= “Graduate or professional school after college" | NA |
| Father Education | What is the highest level of schooling your father completed? | Same as above | NA |
| Hours alone after school | On average, how much time do you spend after school each day at home with no adult present? (Count the hours between the end of school and when you go to bed.) | 1="None or almost none" to 6="More than 5 hours" | Values re-coded (e.g., “None or almost none " was coded as 0 hours) |
