# Supplementary material for: Electronic device use and beverage related sugar and caffeine intake in US adolescents
Source: PLoS One. 2019 Oct 22;14(10):e0223912. doi: 10.1371/journal.pone.0223912 (PMC6805001; doi:10.1371/journal.pone.0223912)
Supplement: S2 Table — (DOCX) [file pone.0223912.s002.docx]

**S2 Table.** Estimated weights and respective caffeine and sugar intake according to age and sex.

|  | **Male** | | **Female** | |
| --- | --- | --- | --- | --- |
|  | **8^th^ Grade** | **10^th^ Grade** | **8^th^ Grade** | **10^th^ Grade** |
| Estimated Ages (years) | 13-14 | 15-16 | 13-14 | 15-16 |
| Estimated Weights (kg) | 48.5 | 59 | 47.5 | 53 |
| Estimated Daily Caffeine Limit (mg/day) | 121.25 | 147.50 | 118.75 | 132.50 |
| Daily Calories for Moderate Activity (calories) | 2300 | 2700 | 2000 | 2000 |
| Strongly Recommended Sugar Limit (cal/day) | 230 | 270 | 200 | 200 |
| Conditionally Recommended Sugar Limit (cal/day) | 115 | 135 | 100 | 100 |
